# Upregulation of miR-181s reverses mesenchymal transition by targeting KPNA4 in glioblastoma

**DOI:** 10.1038/srep13072

**Published:** 2015-08-18

**Authors:** Hongjun Wang, Tao Tao, Wei Yan, Yan Feng, Yongzhi Wang, Jinquan Cai, Yongping You, Tao Jiang, Chuanlu Jiang

**Affiliations:** 1 Department of Neurosurgery, the Second Affiliated Hospital of Harbin Medical University, Harbin, China; 2 Department of Neurosurgery, The First Affiliated Hospital of Nanjing Medical University, Nanjing, China; 3 Department of Neurosurgery, Beijing Tiantan Hospital, Capital Medical University, Beijing, China; 4 Beijing Neurosurgical Institute, Beijing, China; 5 Chinese Glioma Cooperative Group (CGCG).; 6 Department of Urology, Affiliated Zhongda Hospital, Southeast University, Nanjing, China; Surgical Research Center, Medical School, Southeast University, Nanjing, China

## Abstract

The goal of this work was to explore the most effective miRNAs affecting glioblastoma multiforme (GBM) phenotype transition and malignant progression. We annotated 491 TCGA samples’ miRNA expression profiles according to their mRNA-based subtypes and found that the mesenchymal tumors had significantly decreased miR-181 family expression compared with the other three subtypes while the proneural subtype harbored extremely high miR-181 family expression. Patients with high miR-181 family expression had longer overall survival (p = 0.0031). We also confirmed that NF-κB-targeting genes and the EMT (epithelial-mesenchymal transition) pathway were inversely correlated with miR-181 family expression and that the entire miR-181 family inhibited glioma cell invasion and proliferation; of these, miR-181b was the most effective suppressor. Furthermore, miR-181b was validated to suppress EMT by targeting KPNA4 and was associated with survival outcome in the TCGA and CGGA datasets and in another independent cohort. The EMT-inhibitory effect of miR-181b was lost after KPNA4 expression was restored. We also identified the antitumorigenic activity of miR-181b *in vitro* and *in vivo*. Our results showed that miR-181 family expression was closely correlated with TCGA subtypes and patients’ overall survival, indicating that miR-181b, a tumor-suppressive miRNA, could be a novel therapeutic candidate for treating gliomas.

Recently, several advantages have been discovered for the use of miRNAs instead of mRNAs as biomarkers^1^,^2^,^3^. There are nearly 1,000 miRNAs and more than 40,000 protein-coding genes in the human genome. Consequently, genome-wide gene expression data are much more extensive than miRNA expression data. It is more feasible to explore reliable miRNA biomarkers from genome-wide miRNA expression data than from genome-wide gene expression data. Furthermore, miRNAs are relatively stable and are therefore more amenable to analysis in formalin-fixed, paraffin-embedded tissues.

Glioblastoma multiforme (GBM) is the most common form of primary brain cancer. The median survival of primary GBM patients is approximately 1 year, but survival varies widely from <1 week to >3 years after diagnosis^4^. GBM has been classified into four subtypes—proneural, neural, classical, and mesenchymal—by Verhaak *et al*^5^. These four subtypes have different biological behaviors and distinct markers. The contribution of miRNAs to molecular subtype has not been clarified previously. We annotated the miRNA microarrays of 491 TCGA samples by considering the four mRNA subtypes of GBM and analyzed two databases from America (TCGA) and China (CGGA), which together include nearly 700 samples. We found that extremely low miR-181 family expression was a hallmark for the mesenchymal subtype of glioblastomas.

miR-181 family members have been observed in several primary human cancers, including gastric, lung, and prostate cancer, astrocytic tumors, acute myeloid leukemia, and chronic lymphocytic leukemia^6^,^7^,^8^,^9^,^10^,^11^. Low expression of miRNA-181 family members is associated with a poor prognosis in several cancer subtypes^9^,^11^,^12^,^13^,^14^, consistent with our results in both TCGA and CGGA databases. Sun *et al.* demonstrated that miR-181b regulated NF-κB–mediated endothelial cell (EC) activation and vascular inflammation in response to proinflammatory stimuli by targeting KPNA4 and that the rescue of miR-181b expression could provide a new target for anti-inflammatory therapy^15^. Base on the inhibitory effect of miR-181b on NF-κB, we found that miR-181b overexpression could reverse the epithelial-mesenchymal transition (EMT) and inhibit tumor growth *in vitro* and *in vivo*.

## Results

### Aberrant expression of the miR-181 family correlated with TCGA subtypes and prognosis of patients with GBMs

**Figure 1:**
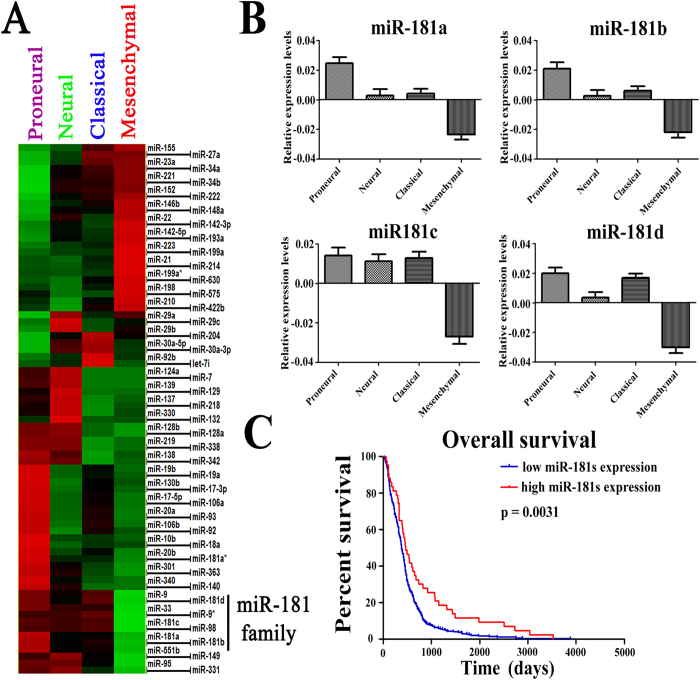
miR-181 family was preferentially expressed in proneural glioma. (**A)** After annotating the miRNA microarrays according to the TCGA mRNA microarray subtypes, we identified the differentially expressed miRNAs and observed that miR-181 family expression was dominant in the proneural subtype. (**B**) There was a significant difference in miR-181 family expression among the four TCGA subtypes. miR-181 family expression in the proneural subtype was significantly higher than in the other three subtypes, especially the mesenchymal subtype. (**C**) When the entire miR-181 family was considered, patients in the miR-181 family low-expression group had a significantly shorter survival time (p = 0.0031).

We annotated miRNA microarrays (Supplementary Figure 1) by considering the four mRNA-based subtypes of glioblastoma according to the TCGA (Supplementary Figure 2). By analyzing the miRNA expression in the four subtypes, we determined that the miR-181 family (miR-181a, miR-181b, miR-181c, and miR-181d) was significantly down-regulated in the mesenchymal subtype compared with the other three subtypes, especially, the proneural subtype (Fig. 1A,B). The expression of the miR-181 family divided patients into two groups according to the expression of miR-181s. Patients in the miR-181 low-expression group had significantly shorter overall survival than those in the miR-181 high-expression group (p = 0.0031) (Fig. 1C). These data supported the hypothesis that miR-181 family members are tumor suppressors in glioma.

### miR-181 family overexpression inhibited cell invasion and proliferation

**Figure 2:**
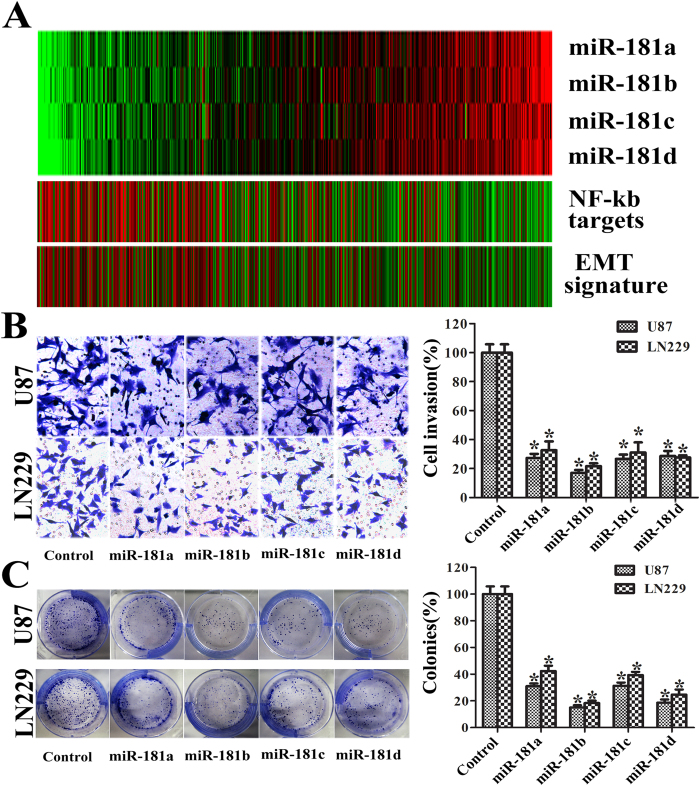
miR-181 family overexpression inhibited cell invasion and proliferation. (**A)** Gene Set Variation Analysis indicated that NF-κB targets and the EMT gene signature were significantly enriched in the tumors with low expression of the miR-181 family. (**B/C)** U87 and LN229 cells were transfected with hsa-miR-181a, hsa-miR-181b, hsa-miR-181c, or hsa-miR-181d expression vectors. Transwell invasion assays revealed that cells transfected with miR-181 family member mimics all had weaker invasion capacity (*p < 0.05). Colony formation assays showed that treated cells exhibited a significant reduction of colony formation after 2 weeks of miR-181 family mimic treatment (*p < 0.05). miR-181b had a trend of more effective suppression on tumor cells invasion and colony formation.

We performed Gene set variation analysis (GSVA) of miR-181 expression to determine the biological functions of the miR-181 family in glioma (Fig. 2A). NF-κB targets and an EMT gene signature list were significantly inversely correlated with miR-181 family expression, and the NF-κB targets and the EMT gene signature list were surprisingly similar. To further determine whether the miR-181 family was associated with glioma invasion, we used two common glioma cell lines U87 and LN229 and observed that invasion and proliferation decreased in cells transfected with miR-181 family members compared with control cells, especially in those transfected with miR-181b (Fig. 2B,C). These results indicated that miR-181 family overexpression participated in the regulation of glioma cell motility and proliferation *in vitro* and that miR-181b was the most effective member.

### Patients with elevated miR-181b expression had a better prognosis because of KPNA4 inhibition and the reversal of EMT

**Figure 3:**
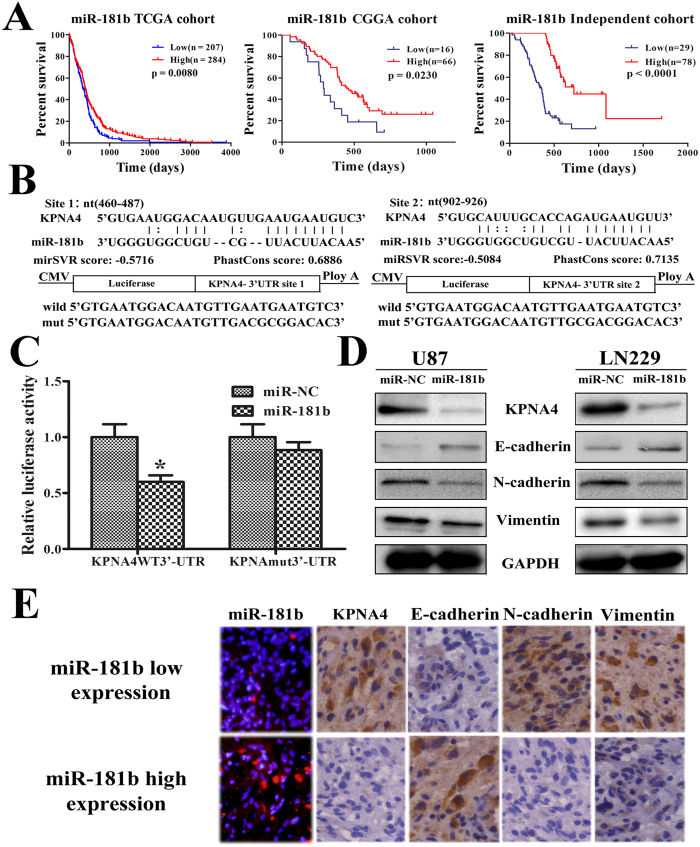
miR-181b could reverse EMT by targeting KPNA4, and its expression correlates with better prognosis. (**A)** In the TCGA and CGGA cohorts and another independent cohort, patients with elevated miR-181b expression had a better prognosis (p = 0.008, p = 0.023, and p < 0.0001, respectively). (**B)** Diagram of the seed sequence of miR-181b matching the 3′UTRs of the KPNA4 gene and the design of wild-type or mutant KPNA4 3′UTR-containing reporter constructs. (**C**) Luciferase reporter assays in glioma cells after co-transfection of cells with wild-type or mutant 3′UTR KPNA and miR-181b. The data represent the fold change in the expression (mean ± SE) of 3 replicates. *p < 0.05. (**D**) Western blot for KPNA4, N-cadherin, E-cadherin, and Vimentin expression 48 hours after transfection with miR-NC or miR-181b. (**E**) Immunohistochemisty revealed that tumors in the miR-181b low expression group had lower KPNA4, N-cadherin, and Vimentin and higher E-cadherin expression.

A survival analysis of 491 GBM miRNA microarray samples from the TCGA indicated that high-grade glioma patients with higher miR-181b expression had a significantly longer OS (p = 0.0080) than those with low expression (Fig. 3A). Similar results were found in the CGGA dataset (82 samples, p = 0.0230) and in another independent cohort (107 samples, p < 0.0001). We performed a bioinformatics search for potential targets of miR-181b (http://www.microrna.org/) to better understand its mechanism in glioma. There are two predicted binding sites for miR-181b in the KPNA4 DNA sequence (Fig. 3B). A luciferase assay was performed in a pMIR-REPORT miRNA reporter vector containing the putative wild-type (WT) and mutant (Mut) KPNA4 3′UTR binding sites. The overexpression of miR-181b inhibited wild-type, but not mutant, KPNA4 reporter activity, suggesting that miR-181b specifically targeted the 3′ UTR of KPNA4 (Fig. 3C).

Compared with the stable expression of miR-NC in U87 and LN229 cells, the stable expression of miR-181b reduced KPNA4 protein expression (Fig. 3D), significantly decreased the expression of the mesenchymal markers (N-cadherin and Vimentin), and increased the expression of the epithelial marker, E-cadherin. These data suggested that the tumor suppressor activity of miR-181b in glioma cells regulated EMT pathways. KPNA4 protein expression in glioma tissues was analyzed by immunohistochemistry (IHC) to determine whether it correlated with reduced miR-181b expression. miR-181b expression was negatively correlated with mesenchymal markers, such as N-cadherin and Vimentin; however, miR-181b expression was positively correlated with the epithelial marker E-cadherin (Table 1) (Fig. 3E). We performed a univariate Cox regression analysis of the independent cohorts using clinical and genetic variables and demonstrated that miR-181b expression, the extent of tumor resection, the preoperative KPS score, and the IDH1 mutation status were significantly associated with OS; however, the variables sex, age, and MGMT promoter methylation status were not associated with OS (Table 2). A multivariate Cox regression analysis of the independent cohorts indicated that miR-181b was an independent prognostic factor (OS: HR, 0.264; 95% CI, 0.144–0.484; p < 0.001).

### Restoring KPNA4 expression counteracted miR-181b overexpression

**Figure 4:**
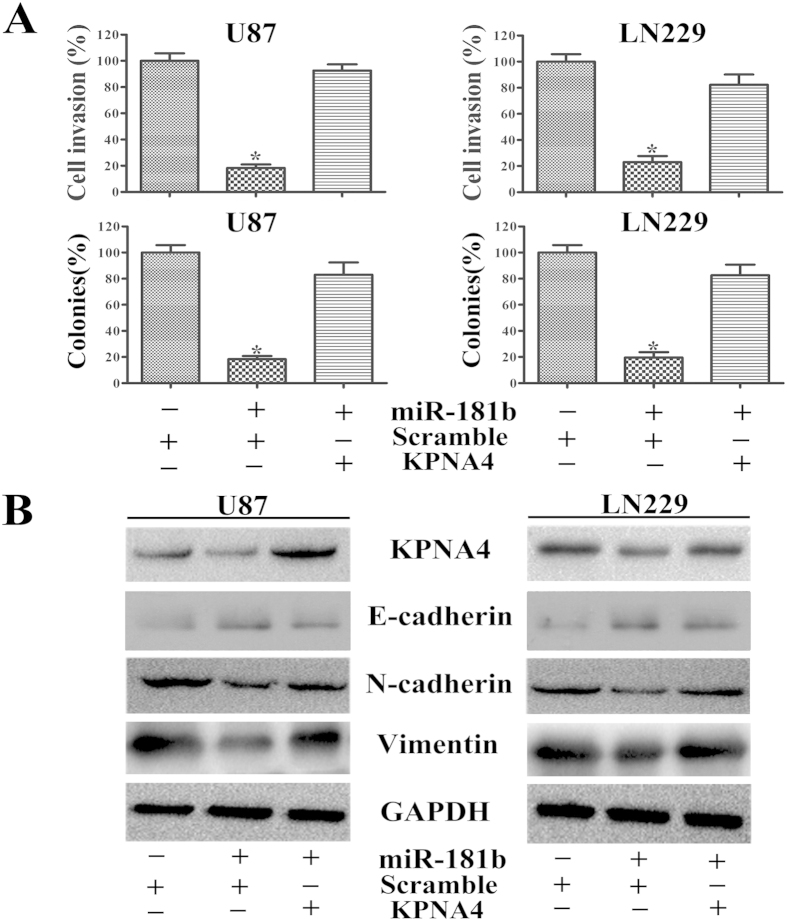
KPNA4 expression overcomes miR-181b-mediated EMT repression. (**A**) Cell invasion and colony formation were inhibited by pReceiver-Lv105-KPNA4, which reversed the effects of miR-181b. (**B**) Western blotting indicated that glioma cells transfected with KPNA4 expressed higher N-cadherin and Vimentin. GAPDH was used as a control. *p < 0.05.

We tested whether KPNA4 was an important target of miR-181b in controlling cell proliferation and invasion and discovered that cell proliferation and invasion were increased in U87/miR-181b and LN229/miR-181b cells transfected with KPNA4 (Fig. 4A). As we confirmed, invasion and proliferation were inhibited by overexpressing miR-181b, which targeted KPNA4. To determine whether miR-181b could directly down-regulate KPNA4 expression and its downstream pathways, we transfected pReceiver-Lv105-KPNA4 into U87 and LN229 cells stably expressing miR-181b or miR-NC. KPNA4 downregulation by miR-181b was counteracted by the overexpression of KPNA4 as evidenced by Western blotting (Fig. 4B). Interestingly, the downregulation of N-cadherin and Vimentin, which was thought to be an indirect result of miR-181b overexpression, was rescued by the upregulation of KPNA4. Similarly, the overexpression of KPNA4 in miR-181b-treated cells down-regulated E-cadherin expression (Fig. 4B).

### miR-181b suppressed glioma tumor growth in orthotopic models

**Figure 5:**
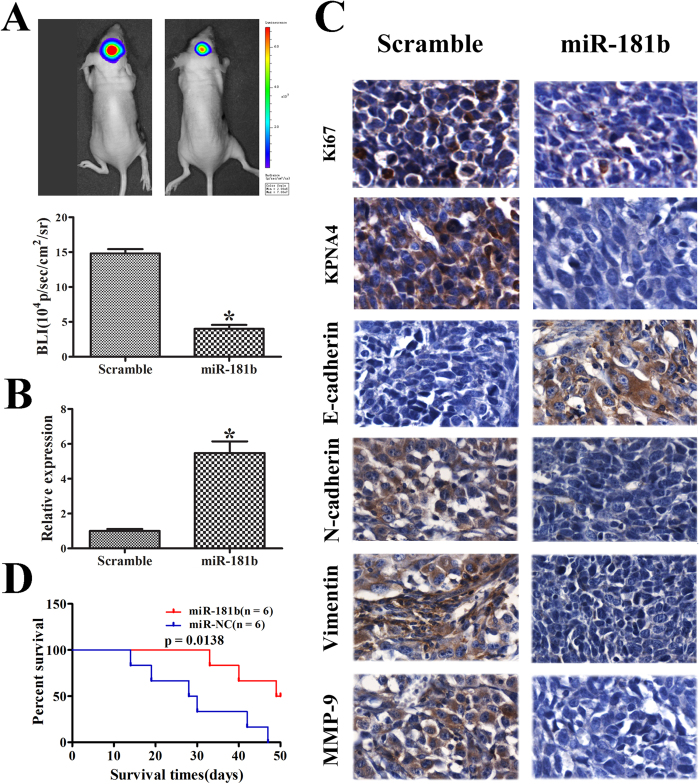
miR-181b overexpression suppressed tumor growth *in vivo*. (**A**) Luminescence imaging was performed on miR-181b-treated LN229 tumors and scramble-treated controls. miR-181b mimic-treated cells displayed a marked reduction in tumor size. (**B**) Kaplan-Meier survival curves indicating that mice transfected with miR-181b showed a significantly better outcome than the miR-NC-treated group (p = 0.0138). (**C**) Real-time PCR revealed that miR-181b expression in the miR-181b-treated group was higher than that in the scramble-treated group. (**D**) Immunohistochemistry for Ki-67, KPNA4, E-cadherin, N-cadherin, Vimentin, and MMP-9 was performed on orthotopic tumor sections. The error bars represent the means ± SD for three independent experiments. *p < 0.05.

An *in vivo* model was established to investigate the relationship between miR-181b and glioma growth. LN229 cells stably expressing miR-181b or miR-NC were injected stereotactically into the brains of nude mice. miR-181b-treated LN229 cells displayed a marked reduction of tumor volume (Fig. 5A). The KPNA4 protein levels were significantly lower in xenografts from miR-181b-expressing cells than in those from miR-NC control cells (Fig. 5B), confirming that miR-181b overexpression suppressed KPNA4 expression *in vivo*. Moreover, the expression levels of Ki-67, N-cadherin, Vimentin, and MMP-9 decreased, whereas E-cadherin expression increased in the miR-181b group relative to the miR-NC group (Fig. 5C). To further evaluate the therapeutic effect of miR-181b on nude mice, the survival period of each group was analyzed by a Kaplan–Meier curve. The miR-181b mimic-treated group showed a significant improvement in survival compared with the control group until the end of the observation period. Only three mice in the miR-181b treatment group died by day 49, whereas no mice were alive in the miR-NC treatment group at this time point (p = 0.013) (Fig. 5D).

## Discussion

miR-181 family members have been reported to be tumor suppressors in glioma^16^,^17^,^18^,^19^,^20^, but there have been no reports on the role of the miR-181 family as a whole. The aberrant expression of the entire miR-181 family is highly correlated with the prognosis and progression of glioma. In the present study, we investigated the expression of the miR-181 family in two independent cohorts comprising nearly 700 glioblastoma patients. We determined that the expression of the miR-181 family was down-regulated in the mesenchymal subtype of GBM compared with other subtypes, suggesting that the miR-181 family exhibited subtype preferences. The increased expression of the miR-181 family conferred a better prognosis and could be utilized as a prognostic indicator. miR-181b was the most effective member of the miR-181 family according to the functional assays in U87 and LN229 cells. The pathway analyses indicated that miR-181b inhibited EMT by blocking KPNA4 expression, which limited glioma growth *in vitro* and *in vivo*.

From a biomarker perspective, our findings suggested novel discovery strategies. First, it is known that each miRNA modulates the expression of hundreds of gene transcripts^21^. The resulting phenotype is likely the aggregate effect of numerous altered transcripts. In this context, we postulated that the miR-181 family collectively regulated other gene transcripts that contributed to the prognostic or predictive value of the miR-181 family; it would be of great interest to identify these transcripts. It is interesting that KPNA4 was the common target of all four members of the miR-181 family according to the bioinformatic prediction and literature. This finding suggested a cooperative effect of the miR-181 family on the malignant progression of glioma. Sun *et al.* reported that miR-181b served as a potent regulator of downstream NF-κB signaling in the vascular endothelium by targeting KPNA4, and they identified the correlated pathways in glioma cell lines via functional assays^15^. However, the expression levels of the miR-181 family members in gliomas are not reported, and their underlying contribution to survival remains to be further investigated.

KPNA4 (importin α3) and importin α4 are the primary importin α isoforms that mediate NF-κB p50/p65 heterodimer translocation into the nucleus^22^. KPNA4 directly binds to the nuclear localization sequences (NLSs) in NF-κB p50 and p65. The NF-κB dimer is held in an inactive state in the cytoplasm by an inhibitory protein (IKB) that masks the NLSs in the subunits. The NLSs in p50 and p65 must be unmasked to enable transport into the nucleus by KPNA4, and the dimers can then activate NF-κB-responsive genes. NF-κB is essential for the epithelial-mesenchymal transition (EMT)^23^; in many cancer types, the loss of E-cadherin coincides with a gain in the expression of the mesenchymal cadherin, N-cadherin. This “cadherin switch” is a hallmark of EMT^24^, which is considered to be the most important malignant process in glioma. The suppression of metastasis via the inhibition of NF-κB activity has been reported for numerous human cancers^25^,^26^,^27^. NF-κB activity is necessary for cells to remain in the mesenchymal state, whereas inhibiting NF-κB reverses EMT, resulting in viable and healthy epithelial cells. These findings provide mechanistic insights into the role of NF-κB in late-stage mammary tumorigenesis and metastasis. Several NF-κB target genes have been induced during EMT, such as those encoding various MMPs; in the present study, we determined that miR-181b overexpression reduced MMP-9 expression (Fig. 5C).

## Materials and Methods

### Patients and samples

Paired miRNA and mRNA profiling data were downloaded from the TCGA data portal (http://cancergenome.nih.gov) and utilized as a discovery set. A total of 491 TCGA glioblastoma samples were included in our study^28^. This method has been used previously to identify the integrated miR-181 family expression^5^. Gene expression subtypes were assigned using the Verhaak *et al.* 840-gene classifier. The dataset from the Chinese Glioma Genome Atlas (CGGA) (http://www.cgga.org.cn), which includes 189 glioblastoma samples (82 samples were used for miRNA microarrays and 107 samples were used for checking miR-181b expression), was obtained as a validation set. The microarray data set was deposited in the Gene Expression Omnibus (GEO) (accession number GSE25632) according to “minimum information about a microarray experiment” (MIAME) guidelines. Written informed consent was obtained from the patients for the publication of this report and any accompanying images. This study was performed with the approval of the Ethics Committee of Capital Medical University and was in compliance with the Helsinki Declaration. Methods were performed in accordance with the approved guidelines.

### GSVA of miR-181 family expression

GSVA of miR-181 family expression was performed using the GSVA package in R. A list of NF-κB target genes was obtained from a published article (Supplementary Table 1)^29^. The EMT gene signature list was obtained from another article (Supplementary Table 2)^30^.

### Real-time PCR and Western blotting

Real-time PCR and Western blotting were performed according to the manufacturer’s instructions as previously described^31^,^32^. The following primers were used: miR-181b sense, 5′-ACACTCCAGCTGGGAACATTCATTGCTGTCGG-3′; miR-181b anti-sense, 5′-TGGTGTCGTG GAGTCG-3′; U6 RT, 5′-TGGTGTCGTGGAGTCG-3′; U6 sense, 5′-CTCGCTTCGGCAGCACA-3′; U6 anti-sense, 5′-AACGCTTCACGAATTTGCGT-3′. qRT-PCR was performed using SYBR Premix DimerEraser (Takara, Dalian, China) on a 7900HT system. U6 levels were used as an internal control, and fold changes were calculated by relative quantification (2^−ΔΔCt^). For Western blotting, the density of specific protein bands was quantified after normalization to the density of the GAPDH band in the same sample.

### Fluorescence *in situ* hybridization, immunohistochemistry, and cell biological assays

Fluorescence *in situ* hybridization (FISH) was utilized to detect miR-181b as previously described^33^. The colony formation and transwell invasion assays have been previously described^34^. Immunohistochemistry was performed using antibodies against E-cadherin (1:100 dilution; Cell Signaling Technology), N-cadherin (1:100 dilution; Cell Signaling Technology), Vimentin (1:250 dilution; Abcam), Ki67 (1:200 dilution; Abcam), and MMP9 (1:100 dilution; Abcam). The degree of immunostaining of sections was viewed and scored separately by two independent investigators. The scores were determined by combining the proportion of positively stained tumor cells and the intensity of staining. The proportion of positively stained tumor cells was graded as follows: 0, no positive tumor cells; 1, < 5% positive tumor cells; 2, 5–20% positive tumor cells; and 3, > 20% positive tumor cells. The intensity of staining was recorded on a scale of 0 (no staining), 1 (weak staining, light yellow), 2 (moderate staining, yellowish brown), or 3 (strong staining, brown). The staining index was calculated as follows: staining index = staining intensity x proportion of positively stained tumor cells. High expression was defined as a staining index score ≥4, and low expression was defined as a staining index <4.

### Luciferase reporter assay

For the luciferase reporter assay, the 3′ UTR of KPNA4 was PCR-amplified from human cDNA. To create a mutant version, the sequence complementary to binding site 1 in the 3′ UTR of miR-181b (AATGAATGA) was replaced with ACGCGGACA, and site 2 (ATGAATGT) was replaced with GACGGACA. The PCR products were digested with SacI and HindIII and inserted into the pMIR-REPORTER vector. The constructs were validated by sequencing. Cells were seeded into a 24-well plate and co-transfected with the wild-type or mutated KPNA4 3′UTR reporter plasmid, pRL-TK, and miR-181b or miR-NC. Luciferase assays were performed 24 h after transfection using the Dual Luciferase Reporter Assay System (Promega, Madison, WI, USA).

### Oligonucleotides and cell transfection

miR-181a, miR-181b, miR-181c, and miR-181d mimics and KPNA4 wild-type and mutant plasmids were chemically synthesized by GenePharma Co., Ltd. (Shanghai, China). Cells at 50–70% confluence were transfected using Lipofectamine (Invitrogen, Carlsbad, CA, USA). Oligonucleotides were transfected into U87 and LN229 glioma cells (purchased from the Institute of Biochemistry and Cell Biology, Chinese Academy of Science, Shanghai, China) at a final concentration of 50 nM as previously described^35^.

### Lentivirus packaging and establishing stable cell lines

A lentiviral packaging kit was purchased from Open Biosystems (Huntsville, AL, USA). Lentiviruses carrying hsa-miR-181b or hsa-miR-negative control (miR-NC) were packaged according to the manufacturer’s protocol. Stable U87 and LN229 cell lines were established by lentiviral infection and puromycin selection. The packaging steps were performed as previously described^31^.

### Orthotopic nude mouse models and treatment

A nude mouse tumor xenograft model was established as previously described^36^,^37^. BALB/c-A nude mice (4 weeks old) were purchased from the Animal Center at the Cancer Institute of the Chinese Academy of Medical Science. Twelve mice were randomly divided into two groups. To establish intracranial gliomas, 0.5 × 10^5^ LN229 glioblastoma cells transduced with luciferase lentivirus and stably expressing miR-181b or miR-NC were implanted stereotactically^38^. Overall survival time of the mice was monitored. After being observed for 50 days, the mice bearing xenograft tumors were sacrificed; the tumor tissues were removed and fixed in formalin, and paraffin-embedded sections were prepared for immunohistochemical analysis.

### Statistical analysis

We applied prediction analysis of microarrays (PAM) as previously reported for the molecular subtype annotation of the four datasets^39^. The integrated miR-181 family expression for predicting survival was developed based on a linear combination of miRNA expression weighted by the regression coefficient derived from the univariate Cox regression analysis. Quantitative results are presented as the means ± standard deviation. The differences in miR-181 family expression in the microarray data were analyzed by Student’s t-test. The survival curves for patients with high or low expression of the miR-181 family were calculated using the Kaplan-Meier method, and the differences were analyzed via the two-sided log-rank test. A p value <0.05 was considered to be statistically significant. All the data analyses were performed in GraphPad Prism and R.

## Additional Information

**How to cite this article**: Wang, H. *et al.* Upregulation of miR-181s reverses mesenchymal transition by targeting KPNA4 in glioblastoma. *Sci. Rep.* **5**, 13072; doi: 10.1038/srep13072 (2015).

## Supplementary Material

Supplementary Information

## Figures and Tables

**Table 1:** The correlation between miR-181b and EMT markers.

Characteristics	miR-181b expression level of 107 GBM samples(%)			
		**Low**	**High**	**p value**
KPNA4	Low	9/53 (17.0)	46/54 (85.2)	
	High	44/53 (83.0)	8/54 (14.8)	<0.01
E-cadherin	Low	38/53 (71.7)	12/54 (22.2)	
	High	15/53 (28.3)	42/54 (77.8)	<0.01
N-cadherin	Low	12/53 (22.6)	39/54 (72.2)	
	High	41/53 (77.4)	15/54 (17.8)	<0.01
Vimentin	Low	15/53 (28.3)	37/54 (68.5)	
	High	38/53 (71.7)	17/54 (31.5)	<0.01

**Table 2:** Cox Hazard Regression Analyses of Clinicopathologic Factors and the miR-181b for Overall Survival in the Independent Cohort (n = 107).

Variable	Univariate analysis			Multivariate analysis		
	**HR**	**95% CI**	**p value**	**HR**	**95% CI**	**p value**
Sex	0.864	0.538–1.386	0.544			
Age	1.047	0.650–1.687	0.850			
Preoperative KPS score	0.394	0.242–0.641	<0.001	0.516	0.304–0.875	0.014
Extent of resection	0.491	0.292–0.828	0.008	0.652	0.371–1.145	0.137
IDH1 mutation	0.399	0.216-0.737	0.003	0.320	0.167-0.613	0.001
MGMT promoter methylation	1.031	0.504-2.108	0.933			
miR-181b	0.287	0.172-0.478	<0.001	0.264	0.144-0.484	<0.001

## References

[b1] Calin G. A. & Croce C. M. MicroRNA signatures in human cancers. J. Nat Rev Cancer. 6, 857–866 (2006). 10.1038/nrc1997 17060945

[b2] Volinia S. *et al.* A microRNA expression signature of human solid tumors defines cancer gene targets. J. Proc Natl Acad Sci USA. 103, 2257–2261 (2006). 10.1073/pnas.0510565103 PMC1413718 16461460

[b3] Lim L. P. *et al.* Microarray analysis shows that some microRNAs downregulate large numbers of target mRNAs. J. Nature. 433, 769–773 (2005). 10.1038/nature03315 15685193

[b4] Ohgaki H. *et al.* Genetic pathways to glioblastoma: a population-based study. J. Cancer Res. 64, 6892–6899 (2004). 10.1158/0008-5472.CAN-04-1337 15466178

[b5] Verhaak R. G. *et al.* Integrated genomic analysis identifies clinically relevant subtypes of glioblastoma characterized by abnormalities in PDGFRA, IDH1, EGFR, and NF1. J. Cancer Cell. 17, 98–110 (2010). 10.1016/j.ccr.2009.12.020 PMC2818769 20129251

[b6] Marcucci G. *et al.* Prognostic significance of, and gene and microRNA expression signatures associated with, CEBPA mutations in cytogenetically normal acute myeloid leukemia with high-risk molecular features: a Cancer and Leukemia Group B Study. J. J Clin Oncol. 26, 5078–5087 (2008). 18809607 10.1200/JCO.2008.17.5554 PMC2652095

[b7] Shi L. *et al.* hsa-mir-181a and hsa-mir-181b function as tumor suppressors in human glioma cells. J. Brain Res. 1236, 185–193 (2008). 10.1016/j.brainres.2008.07.085 18710654

[b8] Chen G. *et al.* MicroRNA-181a sensitizes human malignant glioma U87MG cells to radiation by targeting Bcl-2. J. Oncol Rep. 23, 997–1003 (2010). 10.3892/or_00000725 20204284

[b9] Visone R. *et al.* miR-181b is a biomarker of disease progression in chronic lymphocytic leukemia. J. Blood. 118, 3072–3079 (2011). 10.1182/blood-2011-01-333484 PMC3175784 21636858

[b10] Zhi F. *et al.* The use of hsa-miR-21, hsa-miR-181b and hsa-miR-106a as prognostic indicators of astrocytoma. J. Eur J Cancer. 46, 1640–1649 (2010). 20219352 10.1016/j.ejca.2010.02.003

[b11] Schwind S. *et al.* Prognostic significance of expression of a single microRNA, miR-181a, in cytogenetically normal acute myeloid leukemia: a Cancer and Leukemia Group B study. J. J Clin Oncol. 28, 5257–5264 (2010). 21079133 10.1200/JCO.2010.29.2953 PMC3018359

[b12] Slaby O. *et al.* MicroRNA-181 family predicts response to concomitant chemoradiotherapy with temozolomide in glioblastoma patients. J. Neoplasma. 57, 264–269 (2010). 10.4149/neo_2010_03_264 20353279

[b13] Schaefer A. *et al.* Diagnostic and prognostic implications of microRNA profiling in prostate carcinoma. J. Int J Cancer. 126, 1166–1176 (2010). 19676045 10.1002/ijc.24827

[b14] Marcucci G. *et al.* MicroRNA expression in cytogenetically normal acute myeloid leukemia. J. N Engl J Med. 358, 1919–1928 (2008). 18450603 10.1056/NEJMoa074256

[b15] Sun X. *et al.* MicroRNA-181b regulates NF-kappaB-mediated vascular inflammation. J. J Clin Invest. 122, 1973–1990 (2012). 22622040 10.1172/JCI61495 PMC3366408

[b16] Li P. *et al.* MiR-181b suppresses proliferation of and reduces chemoresistance to temozolomide in U87 glioma stem cells. J. J Biomed Res. 24, 436–443 (2010). 23554660 10.1016/S1674-8301(10)60058-9 PMC3596691

[b17] Wang J., Sai K., Chen F. R. & Chen Z. P. miR-181b modulates glioma cell sensitivity to temozolomide by targeting MEK1. J. Cancer Chemother Pharmacol. 72, 147–158 (2013). 10.1007/s00280-013-2180-3 23645289

[b18] Lorenz M. P. *et al.* Ethene adsorption and dehydrogenation on clean and oxygen precovered Ni(111) studied by high resolution x-ray photoelectron spectroscopy. J. J Chem Phys. 133, 014706 (2010). 20614983 10.1063/1.3456732

[b19] Conti A. *et al.* miR-21 and 221 upregulation and miR-181b downregulation in human grade II-IV astrocytic tumors. J. J Neurooncol. 93, 325–332 (2009). 19159078 10.1007/s11060-009-9797-4

[b20] Zhang W. *et al.* miR-181d: a predictive glioblastoma biomarker that downregulates MGMT expression. J. Neuro Oncol. 14, 712–719 (2012). 10.1093/neuonc/nos089 PMC3367855 22570426

[b21] Hendrickson D. G. *et al.* Concordant regulation of translation and mRNA abundance for hundreds of targets of a human microRNA. J. PLoS Biol. 7, e1000238 (2009). 10.1371/journal.pbio.1000238 PMC2766070 19901979

[b22] Fagerlund R., Kinnunen L., Kohler M., Julkunen I. & Melen K. NF-{kappa}B is transported into the nucleus by importin {alpha}3 and importin {alpha}4. J. J Biol Chem. 280, 15942–15951 (2005). 15677444 10.1074/jbc.M500814200

[b23] Huber M. A. *et al.* NF-kappaB is essential for epithelial-mesenchymal transition and metastasis in a model of breast cancer progression. J. J Clin Invest. 114, 569–581 (2004). 15314694 10.1172/JCI21358 PMC503772

[b24] Lehembre F. *et al.* NCAM-induced focal adhesion assembly: a functional switch upon loss of E-cadherin. J. EMBO J. 27, 2603–2615 (2008). 18772882 10.1038/emboj.2008.178 PMC2567408

[b25] Huang S., Pettaway C. A., Uehara H., Bucana C. D. & Fidler I. J. Blockade of NF-kappaB activity in human prostate cancer cells is associated with suppression of angiogenesis, invasion, and metastasis. J. Oncogene. 20, 4188–4197 (2001). 10.1038/sj.onc.1204535 11464285

[b26] Huang S., DeGuzman A., Bucana C. D. & Fidler I. J. Nuclear factor-kappaB activity correlates with growth, angiogenesis, and metastasis of human melanoma cells in nude mice. J. Clin Cancer Res. 6, 2573–2581 (2000). 10873114

[b27] Andela V. B., Schwarz E. M., Puzas J. E., O’Keefe R. J. & Rosier R. N. Tumor metastasis and the reciprocal regulation of prometastatic and antimetastatic factors by nuclear factor kappaB. J. Cancer Res. 60, 6557–6562 (2000). 11118032

[b28] Zhang W. *et al.* Whole-genome microRNA expression profiling identifies a 5-microRNA signature as a prognostic biomarker in Chinese patients with primary glioblastoma multiforme. J. Cancer. 119, 814–824 (2013). 10.1002/cncr.27826 22990979

[b29] Feuerhake F. *et al.* NFkappaB activity, function, and target-gene signatures in primary mediastinal large B-cell lymphoma and diffuse large B-cell lymphoma subtypes. J. Blood. 106, 1392–1399 (2005). 10.1182/blood-2004-12-4901 15870177

[b30] Zarkoob H., Taube J. H., Singh S. K., Mani S. A. & Kohandel M. Investigating the link between molecular subtypes of glioblastoma, epithelial-mesenchymal transition, and CD133 cell surface protein. J. PLoS One. 8, e64169 (2013). 10.1371/journal.pone.0064169 PMC3667082 23734191

[b31] Shi Z. M. *et al.* MiRNA-181b suppresses IGF-1R and functions as a tumor suppressor gene in gliomas. J. RNA. 19, 552–560 (2013). 10.1261/rna.035972.112 PMC3677265 23431408

[b32] Qian X. *et al.* Sequence-dependent synergistic inhibition of human glioma cell lines by combined temozolomide and miR-21 inhibitor gene therapy. J. Mol Pharm. 9, 2636–2645 (2012). 10.1021/mp3002039 22853427

[b33] Liu Y. *et al.* MiR-218 reverses high invasiveness of glioblastoma cells by targeting the oncogenic transcription factor LEF1. J. Oncol Rep. 28, 1013–1021 (2012). 10.3892/or.2012.1902 22766851

[b34] Wang H., Wang Y. & Jiang C. Stromal protein periostin identified as a progression associated and prognostic biomarker in glioma via inducing an invasive and proliferative phenotype. J. Int J Oncol. 42, 1716–1724 (2013). 23467707 10.3892/ijo.2013.1847

[b35] Tong M. *et al.* Rab25 is a tumor suppressor gene with antiangiogenic and anti-invasive activities in esophageal squamous cell carcinoma. J. Cancer Res. 72, 6024–6035 (2012). 10.1158/0008-5472.CAN-12-1269 22991305

[b36] Zhang C. Z. *et al.* MiR-221 and miR-222 target PUMA to induce cell survival in glioblastoma. J. Mol Cancer. 9, 229 (2010). 10.1186/1476-4598-9-229 PMC2939570 20813046

[b37] Zhang J. *et al.* AKT2 expression is associated with glioma malignant progression and required for cell survival and invasion. J. Oncol Rep. 24, 65–72 (2010). 10.3892/or_00000829 20514445

[b38] Shi Z. *et al.* AC1MMYR2, an inhibitor of Dicer-mediated biogenesis of oncomir miR-21, reverses epithelial-mesenchymal transition and suppresses tumor growth and progression. J. Cancer Res. 73, 5519–5531(2013). 10.1158/0008-5472.CAN-13-0280 23811941

[b39] Yan W. *et al.* Molecular classification of gliomas based on whole genome gene expression: a systematic report of 225 samples from the Chinese Glioma Cooperative Group. J. Neuro Oncol. 14, 1432–1440 (2012). 10.1093/neuonc/nos263 PMC3499016 23090983

