# Supplementary material for: Upregulation of miR-181s reverses mesenchymal transition by targeting KPNA4 in glioblastoma
Source: Sci Rep. 2015 Aug 18;5:13072. doi: 10.1038/srep13072 (PMC4539550; doi:10.1038/srep13072)
Supplement: Supplementary Information [file srep13072-s1.pdf]

Upregulation of miR-181s reverses mesenchymal transition by targeting KPNA4 in glioblastoma

Hongjun Wang[a,f,1], Tao Tao[b,1], Wei Yan[c,f], Yan Feng[a,f], Yongzhi Wang[d-f], Jinquan Cai[a,f], Yongping You[c,f,]*, Tao Jiang[d-f,]* and Chuanlu Jiang[a,f,]*

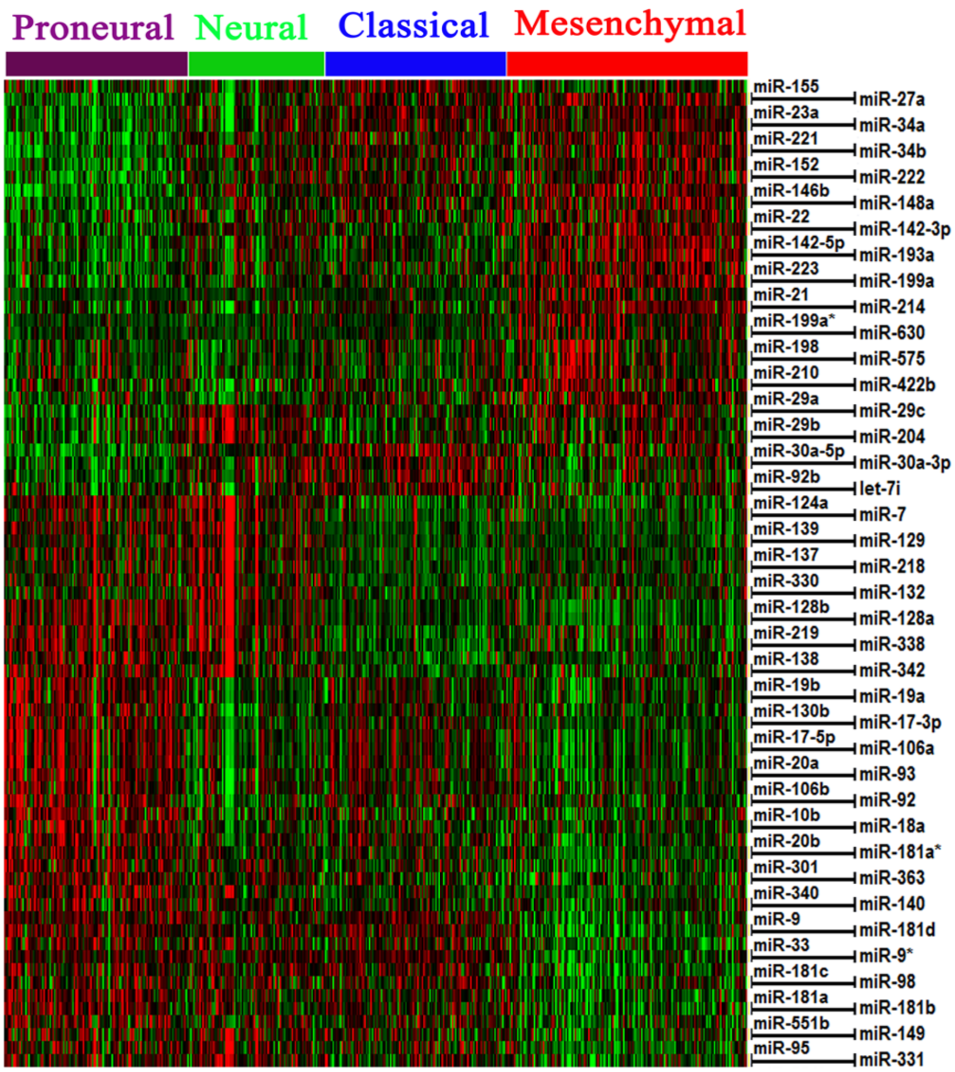

Supplementary Fig.1. GBM subtypes from the TCGA mRNA microarrays. GBM was classified according to 840 representative genes into the following four subtypes: proneural, neural, classical and mesenchymal. The mesenchymal subtype is the most aggressive.

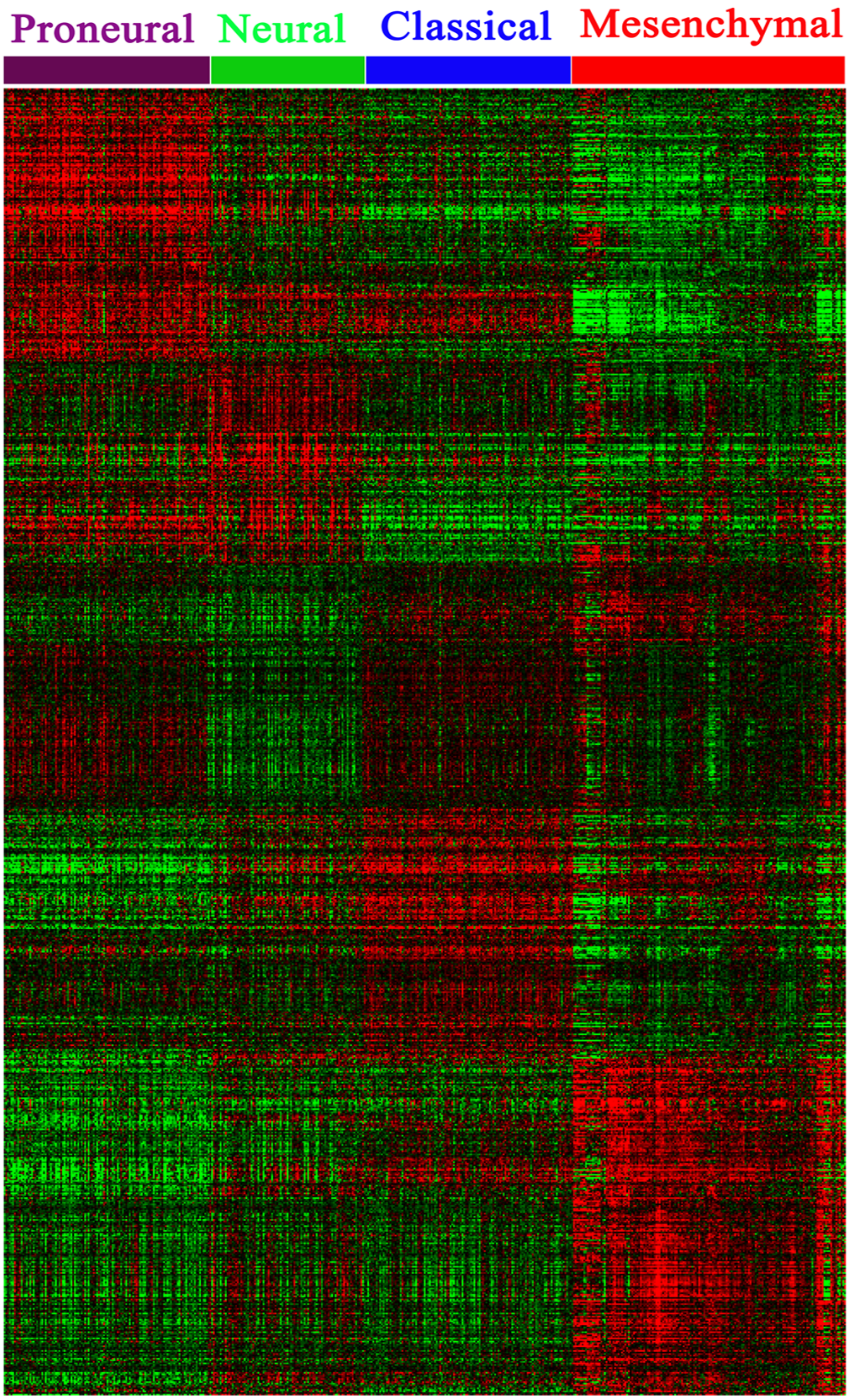

Supplementary Fig.2. GBM subtypes from the TCGA miRNA microarrays. We annotated the TCGA miRNA microarrays and identified four subtypes similar to those obtained from the mRNA microarrays.

Table S1: NFkB Target Gene Sets[1]

	Gene Symbol
**1**	'BCL 2'
**2**	'BCL2A1'
**3**	'BCL2L1'
**4**	'BIRC2'
**5**	'BIRC3'
**6**	'C4orf9'
**7**	'CCL2'
**8**	'CCL22 '
**9**	'CCL3'
**10**	'CCL4'
**11**	'CCND2'
**12**	'CCR7'
**13**	'CD23A'
**14**	'CD44'
**15**	'PRR16'
**16**	'CD69'
**17**	'CD83'
**18**	'CFLAR'
**19**	'DUSP1'
**20**	'DUSP2'
**21**	'EGR1'
**22**	'EMR1'
**23**	'HLA-F'
**24**	'ICAM1'
**25**	'IER2'
**26**	'IER3'
**27**	'IL15RA'
**28**	'IL2RA'
**29**	'I L4RA'
**30**	'IL6'
**31**	'I L8'
**32**	'IRF1'
**33**	'IRF4'
**34**	'JUNB'
**35**	'KAI1'
**36**	'KLF10'
**37**	'LITAF'
**38**	'LSP1'
**39**	'MYB'
**40**	'NCF2'
**41**	'NFkB2'
**42**	'NFKBIA'
**43**	'NK4'
**44**	'PASK'
**45**	'PCAM1'
**46**	'PRKCD'
**47**	'RAFTLIN'
**48**	'REL'
**49**	'RELB'
**50**	'RRAS2'
**51**	'SDC4'
**52**	'SLC2A5'
**53**	'SMAD7'
**54**	'SOD2 '
**55**	'STAT5A'
**56**	'STX4A'
**57**	'TNFAIP3'
**58**	'TNFRSF5'
**59**	'TNFRSF6'
**60**	'TNFSF2'
**61**	'TNIP'
**62**	'TPMT'
**63**	'TRAF1'
**64**	'VIM'
**65**	'WTAP'

References

1. Feuerhake F, Kutok JL, Monti S, et al. NFkappaB activity, function, and target-gene signatures in primary mediastinal large B-cell lymphoma and diffuse large B-cell lymphoma subtypes. *Blood.* 2005;106:1392-1399.

Table S2: Up regulated genes in the genetic signature of EMT[1]

	Gene Symbol
1	'FBLN5'
2	'GREM1'
3	'COL3A1'
4	'COL1A2'
5	'DCN'
6	'CDH2'
7	'ENPP2'
8	'POSTN'
9	'RGS4'
10	'C5ORF13'
11	'PRRX1'
12	'FBN1'
13	'SRGN'
14	'SPOCK1'
15	'PRR16'
16	'DLC1'
17	'BIN1'
18	'RGL1'
19	'IGFBP4'
20	'PVRL3'
21	'CDH11'
22	'OLFML3'
23	'MMP2'
24	'MYL9'
25	'COL5A2'
26	'CTGF'
27	'PLEKHC1'
28	'ZEB1'
29	'ROR1'
30	'PTGER2'
31	'CHN1'
32	'PMP22'
33	'TRAM2'
34	'TAGLN'
35	'TNFAIP6'
36	'CREB3L1'
37	'UGDH'
38	'HAS2'
39	'DNAJB4'
40	'CDKN2C'
41	'CCDC92'
42	'WNT5A'
43	'IGFBP3'
44	'PPM1D'
45	'FILIP1L'
46	'PDGFC'
47	'TBX3'
48	'DPT'
49	'STC1'
50	'LMCD1'
51	'N-PAC /// SEPT6'
52	'NR2F1'
53	'SCCPDH'
54	'MLPH'
55	'LTBP2'
56	'TPM1'
57	'ANKRD25'
58	'DDR2'
59	'SEMA5A'
60	'TGFB1I1'
61	'PCOLCE'
62	'STARD13'
63	'NID1'
64	'SYNC1'
65	'ENOX1'
66	'FSTL1'
67	'VIM'
68	'MME'
69	'C10ORF56'
70	'NRP1'
71	'THY1'
72	'NEBL'
73	'TNS3'
74	'FBLN1'
75	'TUBA1A'
76	'COPZ2'
77	'CYBRD1'
78	'PPAP2B'
79	'PTX3'
80	'FADS2'
81	'BGN /// TSHZ1'
82	'ZBTB38'

References

1. Zarkoob H, Taube JH, Singh SK, Mani SA, Kohandel M. Investigating the link between molecular subtypes of glioblastoma, epithelial-mesenchymal transition, and CD133 cell surface protein. *PLoS One.* 2013;8:e64169.
